# The Enzyme-Mediated Direct Reversal of a Dithymine Photoproduct in Germinating Endospores

**DOI:** 10.3390/ijms140713137

**Published:** 2013-06-25

**Authors:** Linlin Yang, Lei Li

**Affiliations:** 1 Department of Chemistry and Chemical Biology, Indiana University-Purdue University Indianapolis (IUPUI), 402 N Blackford Street, Indianapolis, IN 46202, USA; E-Mail: linlyang@iupui.edu; 2 Department of Biochemistry and Molecular Biology, Indiana University School of Medicine (IUSM), 635 Barnhill Drive, Indianapolis, IN 46202, USA

**Keywords:** thymine dimer, DNA damage, DNA damage repair, radical, transfer pathway

## Abstract

Spore photoproduct lyase (SPL) repairs a special thymine dimer, 5-thyminyl-5,6-dihydrothymine, which is commonly called spore photoproduct, or SP, in germinating endospores. SP is the exclusive DNA photo-damaging product found in endospores; its generation and swift repair by SPL are responsible for the spores’ extremely high UV resistance. Early in vivo studies suggested that SPL utilizes a direct reversal strategy to repair SP in the absence of light. Recently, it has been established that SPL belongs to the radical S-adenosylmethionine (SAM) superfamily. The enzymes in this superfamily utilize a tri-cysteine CXXXCXXC motif to bind a [4Fe-4S] cluster. The cluster provides an electron to the S-adenosylmethionine (SAM) to reductively cleave its C5′-S bond, generating a reactive 5′-deoxyadenosyl (5′-dA) radical. This 5′-dA radical abstracts the proR hydrogen atom from the C6 carbon of SP to initiate the repair process; the resulting SP radical subsequently fragments to generate a putative thymine methyl radical, which accepts a back-donated H atom to yield the repaired TpT. The H atom donor is suggested to be a conserved cysteine141 in *B. subtilis* SPL; the resulting thiyl radical likely interacts with a neighboring tyrosine99 before oxidizing the 5′-dA to 5′-dA radical and, subsequently, regenerating SAM. These findings suggest SPL to be the first enzyme in the large radical SAM superfamily (>44,000 members) to utilize a radical transfer pathway for catalysis; its study should shed light on the mechanistic understanding of the SAM regeneration process in other members of the superfamily.

## 1. The Unique Photo-Damage in Bacterial Endospores

Bacterial endospores are the longest-lived cells known on earth. They are so resistant to harsh environments, such as vacuum, heat, desiccation and irradiation, that they may survive in outer space as hitchhikers clinging to the outside of spacecraft [1]. Due to the high solar irradiation flux, in particular, UV irradiation, the ability to protect their genomic DNA is suggested to be the key to spores’ survival in outer space. Considering the number of deadly diseases associated with the spore forming bacterial strains and the fact that UV irradiation is a common sterilization means used in our daily life [2], understanding the spore UV resistance is of particular significance.

**Figure 1:**
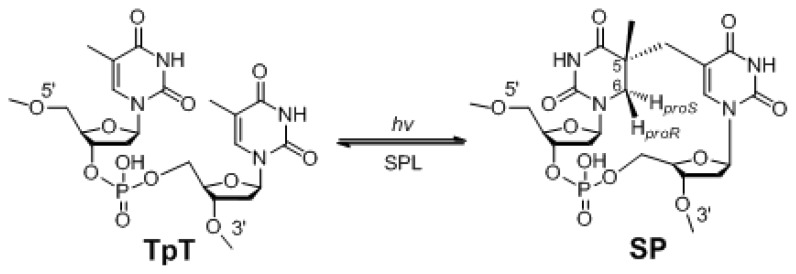
Spore photoproduct (SP) formation under UV irradiation and its repair by spore photoproduct lyase (SPL).

Among the four nucleobases, thymine (T) is the most UV sensitive one [3–5]. In typical cells, the genomic DNA adopts a B-conformation, and thymine photo-dimerization leads to the formation of cyclobutane pyrimidine dimer (CPD) and pyrimidine (6-4) pyrimidone photoproduct ((6-4) PD) [4]. In contrast, in endospores, the genomic DNA adopts an A-conformation, due to the low water content, as well as the binding of small acid-soluble proteins (SASPs) [6–8]. Endospores also contain a large amount of dipicolinic acid (DPA), which makes up 5%–10% of the spore dry weight [9–11]. DPA probably exists in spores as a 1:1 chelate with divalent cations, predominantly Ca^2+^. The Ca-DPA complex is suggested to serve as a photo-sensitizer [11], mediating the triplet-state energy transfer after UV excitation to the thymine bases [10]. Consequently, UV irradiation yields a special thymine dimer, 5-thyminyl-5,6-dihydrothymine (commonly called spore photoproduct, or SP), as the dominant DNA UV lesion (Figure 1). Under mono-chromatic 254 nm UV light, SP accounts for as many as 95% of the total photoproducts in spore’s genomic DNA, and the *cis-syn* CPDs comprise ~5% of the photo-lesions [12]. Spores exposed to broad-spectrum UV, such as sunlight (UVA + UVB), produce more CPDs as “minor” photoproducts [13].

SPs formed under UV irradiation could account for as many as 8% of the total thymine residues in spore genomic DNA [11,14]. After selectively labeling the thymine residues by deuterium, Lin proves that SP is formed via an intramolecular H-atom transfer process, with an H atom being transferred from the -CH_3_ moiety at the 3′-thymine of the TpT step to the H*_6proS_* position at the 5′-thymine in the formed SP (Figure 1) [15]. SPs accumulate in dormant spores. When spores start germinating, they must be repaired, as SPs prove lethal to the germinated bacteria [9,16].

## 2. SP Repair by NER and SPL

The germinating spores utilize two major pathways to repair SP: the general nucleotide excision repair pathway (NER) mediated by the *uvr* genes [17] and a spore-specific DNA repair system, which involves *in situ* monomerization of SP into two thymine residues mediated by an enzyme, named spore photoproduct lyase (SPL, Figure 1), which is encoded by the *spl* gene [18–21]. Blocking either pathway only slightly affects the spores’ UV sensitivity; both pathways have to be interrupted before the spores’ UV resistance is deprived [19,22]. Between these two pathways, the faster repair rate by SPL suggests that it is the major enzyme to repair SP damages in germinating spores [12,19].

SPL is suggested to be expressed during spore formation and packaged in formed spores [23]. It catalyzes the SP repair reaction via a direct reversal strategy with no light needed in germinating spores. Such a repair mechanism is in contrast to that of the DNA photolyases, which utilize the energy from light, and a fully reduced flavin cofactor to reverse CPDs and (6-4) PDs [24,25]. Also, SPL is known to be specific for the repair of SP, and it does not repair CPDs and (6-4) PDs [26,27]. The direct reversal mechanism used by SPL was first indicated by Donnellan and Stafford in 1968 [16], three years after SP was discovered in UV irradiated endospores [28]. After labeling thymine residues by tritium and generating SPs via UV irradiation, the authors found that no tritium was leaked into the media after SP repair in the germinated bacteria [16]. If an excision repair mechanism is involved, one would expect the labeled SP damages to be released into the media. Later, Rupert *et al*. proved that the radioactivity disappearing from SP appeared to be stoichiometrically recovered in thymine [21], further supporting this direct-reversal hypothesis. The property of SP and the strong UV resistance it brings to bacterial spores have been covered by several excellent reviews in the past several years [1,9,10,29–32]. In this paper, we will focus on the latest progress in the mechanistic elucidation of SPL.

## 3. Mechanism of SPL Repair

**Figure 2:**
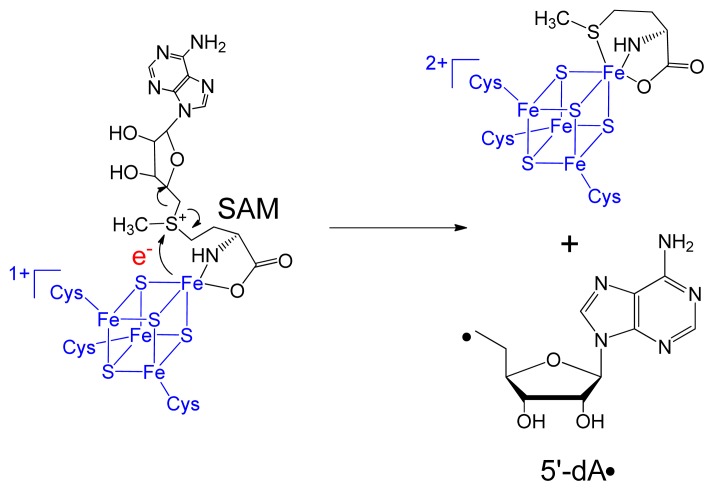
*S*-adenosylmethionine (SAM) reductive cleavage with the electron provided by the [4Fe-4S]^+^ cluster to yield the 5′-dA radical and methionine in radical SAM enzymes.

SPL is a member of the so-called radical *S*-adenosylmethionine (SAM) superfamily, which is defined by the characteristic CXXXCXXC motif [33], although recent evidence suggests that other three-cysteine motifs also facilitate this radical chemistry [34–36]. The three cysteine residues in the CXXXCXXC motif serve as ligands for three irons in the [4Fe-4S] cluster; the fourth iron in the cluster is coordinated by SAM under a bi-dentate manner, with its amino and carboxylate moieties serving as the fourth and fifth ligands (Figure 2) [37]. The cluster at its +1 oxidation state donates an electron to SAM to cleave its C5′-S bond, generating a 5′-deoxyadenosyl radical (5′-dA•).

Currently, 44,680 radical SAM enzymes from more than 3000 species have been identified to utilize this reaction to generate radical species for catalysis [38]. Over 40 distinct reaction types are represented, including glycyl-radical cofactor formation, sulfuration, methylation, methylthiolation, hydroxylation, carbon–carbon bond formation, carbon–carbon bond fragmentation, dehydrogenation, decarboxylation, metallocofactor maturation and complex rearrangements [38,39]. These transformations are found in a myriad of biological processes, including steps in metabolism, DNA/RNA modification and biosynthesis of vitamins, coenzymes and many antibiotics [35,40–52].

**Figure 3:**
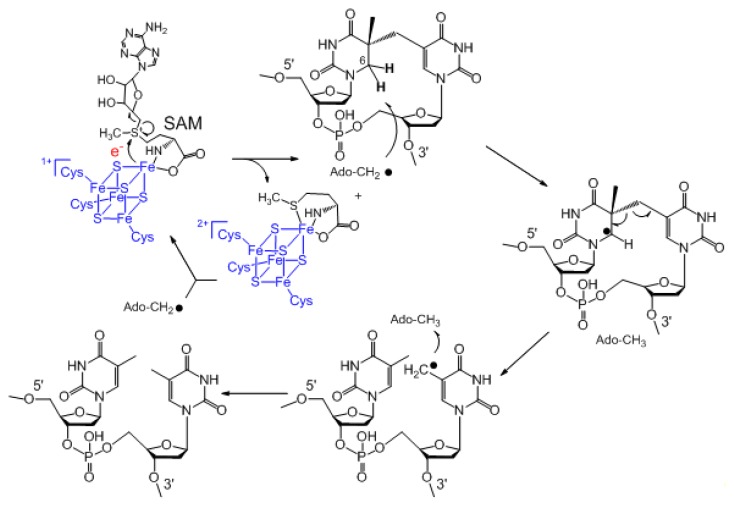
Previously proposed mechanism for the SPL catalyzed SP repair reaction.

SPL was first suggested to be an iron-sulfur enzyme by Rebeil *et al.*, using a radical mechanism similar to that found in the activating enzymes of the class III anaerobic ribonucleotide reductase and pyruvate-formate lyase [53]. Rebeil *et al.* also demonstrated that SAM is cleaved to 5′-dA during the SPL catalyzed SP repair process [54]. Later, using an SP analogue, Mehl and Begley indicated that SPL initiates the SP repair via a hydrogen atom abstraction reaction at the C6 position, which was very likely mediated by the 5′-dA• generated via a SAM cleavage reaction [55]. Cheek and Broderick selectively labeled the thymine residues in a plasmid DNA with tritium and generated the SP dimer via photochemistry; they recovered the tritium-labeled SAM after SPL reaction [56]. Moreover, one molecule of SAM was suggested to catalyze >500 turnovers. These observations were similar to those observed in another radical SAM enzyme—the lysine 2,3-aminomutase [57–60]—suggesting that SAM is regenerated after each catalytic turnover (Figure 3) [56]. However, among all the SPL studies to date, this is the only report supporting SAM to have a truly catalytic role. A stoichiometric or a nearly stoichiometric amount of SAM relative to the repaired SP is needed in all the other studies [26,61–69]. Although it is reasonable that SAM is regenerated during SPL catalysis, future work is needed to firmly establish this assumption.

## 4. The Stereoselectivity of SPL Reaction

As indicated in Figure 1, the C5 position in 5′-thymine of SP is chiral. It was suggested by Begley to adopt an *R* configuration, due to the right-handed helical structure of DNA [70]. However, Frediel *et al*. argued the configuration to be an *S* configuration [71,72]. After producing the dinucleotide SP TpT via TpT photoreaction in a dry film environment [73], the chiral configuration at the C5 center was confirmed to be *R* via 2D NMR spectroscopic characterizations [73]. The SP TpT generated exhibited identical properties to that prepared via photo-irradiation of calf thymus DNA, followed by enzyme digestion [26]. Additionally, only the 5*R*, but not the 5*S* SP (prepared by chemical synthesis), can be repaired by SPL [68], which is further confirmed by the structure of a formacetal linker containing dinucleotide SP T_CH2_T [74]. At last, via X-ray structural studies of the dinucleoside SP containing oligonucleotide, Heil *et al*. showed only the *5R* SP isomer can fit in the right-handed duplex structure; while the *5S* isomer likely causes severe helical disturbance [75]. All these results suggest the 5*R* SP TpT to be the truly biologically relevant species.

Also shown in Figure 1, the C6 position of SP is pre-chiral, possessing two H atoms occupying *proR* and *proS* positions, respectively. Enzyme reactions are highly stereoselective. As the SPL-mediated SP repair reaction is likely initiated by an H abstraction at the C6 carbon [55,56], it is of significance to identify the abstracted H-atom. After selectively labeling thymine residues by deuterium, Lin *et al.* successfully prepared the deuterium labeled dinucleotide SP TpTs after UVC irradiation, with a deuterium occupying the H*_6proR_* and H*_6proS_* positions, respectively [15]. Using these selectively labeled SPs as enzyme substrates, Yang *et al.* proved that the 5′-dA• generated via SAM reductive cleavage reaction takes the H*_6proR_* atom [65].

## 5. SPL Catalysis Requires an Essential Cysteine

The stereochemistry during the SPL catalysis is now established after these disputes. The previous SPL mechanism was also questioned. The mechanism shown in Figure 3 indicates that 5′-dA is involved in both hydrogen atom transfer steps: the 5′-dA• abstracts an H atom at the 5′-thymine of SP; it has to move down for one nucleotide, roughly 3.4 Å in the framework of a regular B-DNA, to donate the H atom back to the methyl radical at the 3′-thymine, yielding the repaired TpT. Theoretical calculations imply that such a movement requires a dramatic protein conformational change and is unfavorable energetically [76,77]. Later, Fajardo-Cavazos *et al.* deleted the original *uvr* and *spl* genes from the *B. subtilis* genome and integrated the engineered *splB* genes carrying different point mutations at the *amyE* locus, respectively [78]. The *B. subtilis* SPL (SPL_(Bs)_) has four conserved cysteine residues, C91, C95, C98 and C141. The first three cysteines are within the radical SAM CXXXCXXC motif; mutating any of these cysteines would drastically destabilize the [4Fe-4S] cluster and deactivate the enzyme [78]. Surprisingly, the C141A mutation equally deactivates SPL, making the corresponding *B. subtilis* endospores carrying this SPL mutant very sensitive toward UV irradiation [78]. This observation suggests that the fourth cysteine must be involved in enzyme catalysis.

**Figure 4:**
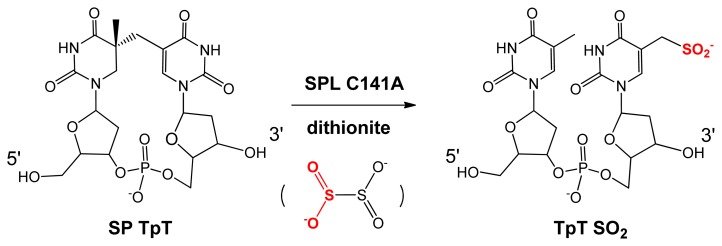
Formation of TpTSO_2_^−^ after SP TpT repair by the *B. subtilis* SPL 141A mutant.

Fontecave *et al.* re-examined this C141A_(Bs)_ mutant in an *in vitro* enzymatic study using dinucleotide SP TpT as substrate [64]. After reducing the [4Fe-4S] cluster from the 2+ to 1+ oxidation state by sodium dithionite to initiate the radical SAM reaction, they identified a TpTSO_2_^−^ species with the SO_2_^−^ moiety attached to the methyl group of the 3′-thymine allylic radical intermediate as the major repair product (Figure 4). The -SO_2_^−^ moiety originated from the homolytic cleavage of the S-S bond in dithionite; the resulting •SO_2_^−^ subsequently combined with the methyl radical to yield TpTSO_2_^−^[79]. Such an observation indicates that the C141A mutation disturbs the H-atom back donation step in the SP repair process.

**Figure 5:**
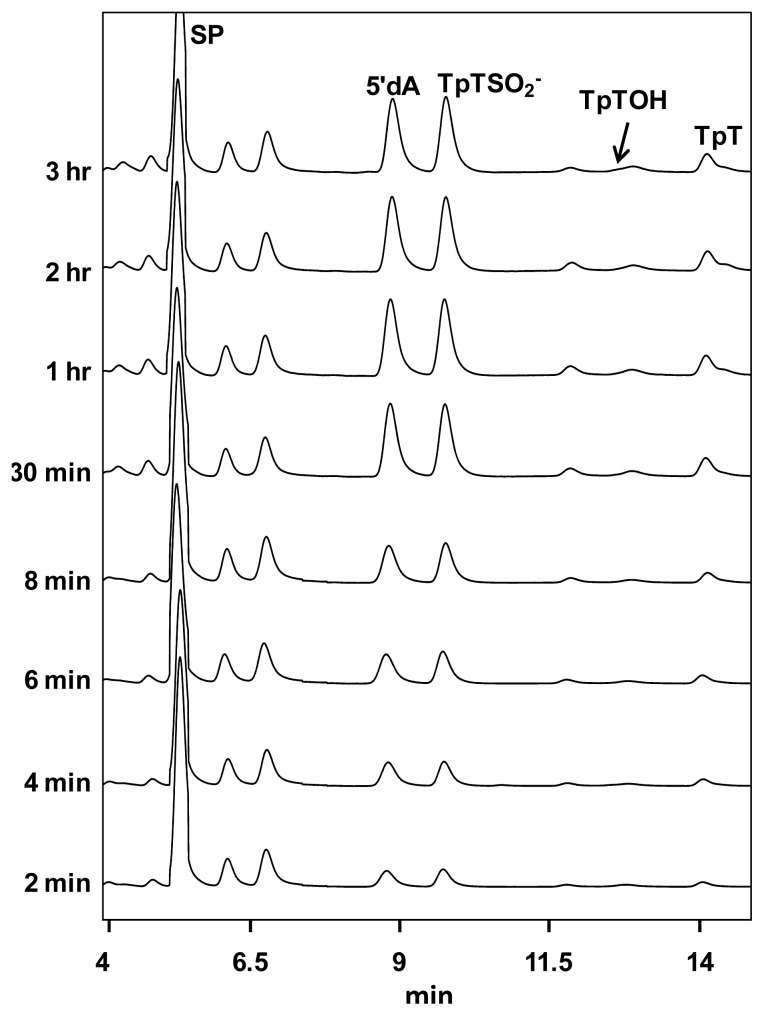
HPLC chromatograph of the SP TpT repair process mediated by the *B. subtilis* SPL C141A mutant with 30 μM enzyme, 150 μM SAM and 1 mM dithionite. Under the HPLC program, the SP TpT was eluted at 5.4 min, 5′-dA at 8.9 min, TpTSO_2_^−^ at 9.8 min, TpTOH at 12.9 min and TpT at 14.1 min. Linear formations of TpTSO_2_^−^ and TpT were observed in the first 30 min of the reaction (the figure is adapted with permission from reference [66]. Copyright (2012) American Chemical Society).

Is the C141 the direct H donor? Besides cysteine, another amino acid commonly involved in a radical reaction is tyrosine. The SPL enzyme has eight conserved tyrosine residues. If a tyrosine residue is located between C141 and the SP substrate and serves as the direct H donor to the thymine methyl radical, it should still be present in the C141A mutant and well-positioned for the H-donation reaction. Therefore, TpT is the expected product during the first enzyme turnover. TpTSO_2_^−^ will not be formed until this tyrosine residue is consumed, resulting in a lag phase for its generation. Yang *et al.* thus examined the kinetics for the C141A_(Bs)_ mutant reaction and found no evidence for the presence of such a lag phase (Figure 5). They then concluded that C141 in *B. subtilis* SPL is very likely to be the direct H donor to the thymine allylic radical [66].

**Figure 6:**
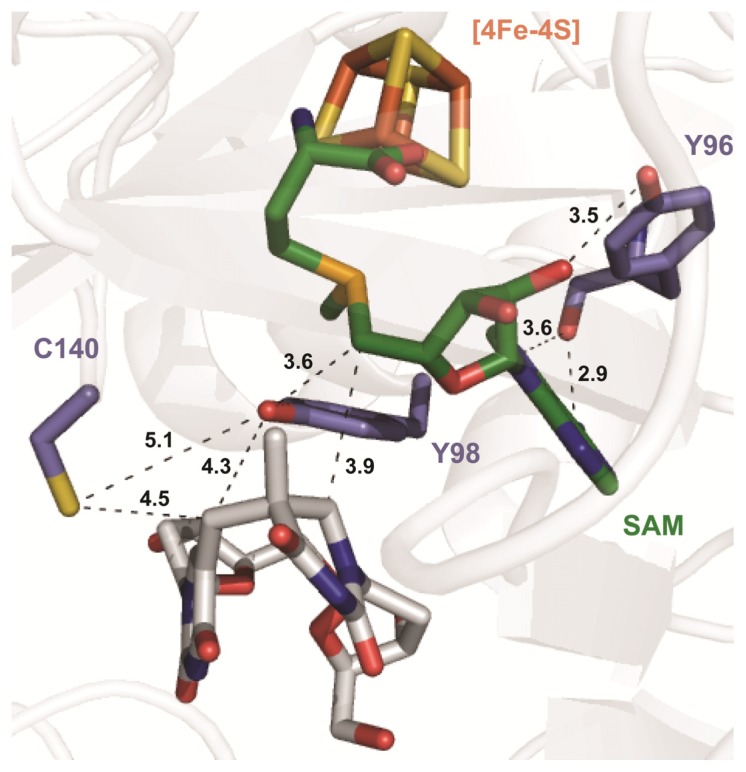
Active site of *Gt* SPL in complex with SP (in white), the [4Fe-4S] cluster and SAM. The C140_(Gt)_, Y96_(Gt)_ and Y98_(Gt)_ residues correspond to the C141_(Bs)_, Y97_(Bs)_ and Y99_(Bs)_ residues in *Bs* SPL, respectively. The distances between protein residues, SP and SAM are indicated by dashed lines (PDB code 4FHD) (the figure is adapted with permission from reference [67]. Copyright (2013) American Chemical Society).

Such a hypothesis is confirmed by a newly solved crystal structure with the SPL originated from the bacterium *Geobacillus thermodenitrificans (Gt)* [62]. The SPL_(Gt)_ shares ~77% sequence identity with the SPL_(Bs)_, but exhibits a −1 sequence shift for the conserved amino acids. As shown in Figure 6, the crystal contained an uncleaved SAM molecule and a dinucleoside SP. The distance between the methylene carbon and the conserved cysteine (C140) was found to be 4.5 Å, which should be even shorter after the SP fragmentation step to generate a thymine methyl radical. This conserved cysteine is thus well-positioned to be the intrinsic H donor, as predicted by the enzymology data.

## 6. The Presence of a Novel Radical Transfer Pathway Mediated by Tyrosine Residues in SPL

After donating the H atom to the thymine allylic radical, the C141 will become a thiyl radical. If SAM is to be regenerated after each catalytic cycle, the thiyl radical must be involved. The C141_(Bs)_ is known to be accessible from the aqueous media [66]. The C141-S• radical is thus likely exposed to the small thiol compounds added in the solution, which may reduce the C141-S• radical and result in an abortive SP repair reaction. However, addition of small thiols, such as β-mercaptoethanol, appears to have no impact on the SP repair process [66], which is hard to achieve if the cysteine is the only protein residue involved in enzyme catalysis, but is possible if the cysteine is a part of a tightly coupled radical transfer pathway. A nearby tyrosine residue (Y98_(Gt)_, Figure 6) is located between this cysteine and the SAM molecule, suggesting it is likely involved in SAM regeneration. Thus, a radical transfer pathway, with the cysteine-tyrosine pair as the essential elements, may exist for the putative SAM regeneration process in SPL.

This hypothesis is supported by recent kinetic isotope effect (KIE) studies [67]. Using deuterium labeled TpT, the H*_6proR_* position in the 5′-T of SP can be labeled after photoreaction [15]. The H*_6proR_* atom is abstracted by 5′-dA• in the SPL catalyzed SP repair reaction. This hydrogen abstraction step is kinetically significant, as indicated by an apparent KIE of 2.8 ± 0.3 [65]. Furthermore, using a mixture of unlabeled and deuterium labeled SPs as substrates, the competitive KIE was determined for the wild-type (WT) SPL_(Bs)_ reaction to be 3.4 ± 0.3 (Table 1). The C141A_(Bs)_ mutant slows the reaction by three-fold; it also reduces the KIEs, due to the slower quenching of the thymine methyl radical [66]. Surprisingly, the Y99F_(Bs)_ mutant exhibits much bigger KIEs, with the apparent KIE to be 10.5 ± 1 and competitive KIE to be 9 ± 1. These enhanced KIEs, comparing with those from the WT SPL_(Bs)_ and C141A_(Bs)_ mutant, suggest that the rate determining step in SPL catalysis is altered by this Y→F mutation [67]. The H-abstraction from the SP substrate likely becomes the new rate determining step in the Y99F_(Bs)_ mutant reaction. In contrast, the H-abstraction step to yield 5′-dA• before SAM regeneration is rate-limiting in the WT SPL reaction [67].

A surprising finding by the enzymology studies is that the Y97F_(Bs)_ mutant also results in a three-fold rate reduction relative to the WT enzyme. Using deuterated SP substrates, the apparent and competitive KIEs were found to be 16 ± 1.5 and 11.5 ± 1.5, respectively, which are comparable to those exhibited by the Y99F_(Bs)_ mutant, but much bigger than those shown by the WT enzyme. According to the structure in Figure 6, such a residue (Y96 of the SPL_(Gt)_) is involved in SAM binding by interacting with the adenosyl ring. The enzyme kinetic data, however, suggest that it may have other functions in SPL catalysis, as well. The current hypothesis is that it may serve as a radical stabilization residue by interacting with the 5′-dA• generated either after SAM reductive cleavage or before SAM regeneration, resulting in a partially populated Y• at this residue. The putative •Y97_(Bs)_ radical can fine-tune the redox potential of the •Y99_(Bs)_ and/or the 5′-dA• by delocalizing the radical intermediate and reducing the energy barrier for the H-abstraction step, making it kinetically competent [67].

**Figure 7:**
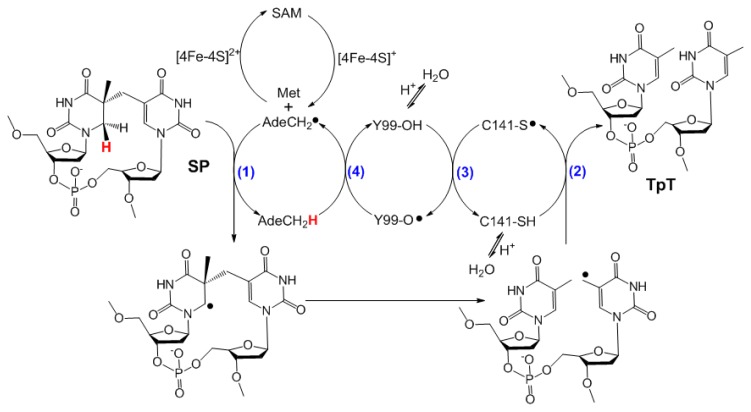
Hypothesized reaction mechanism for SPL (The residues are numbered according to the protein sequence in *Bs* SPL). This mechanism implies that SPL uses a minimum of four H atom transfer (HAT) processes (labeled in blue numbers) in each catalytic cycle. One of the four processes occurs between a tyrosine and a cysteine, suggesting that SPL uses a novel HAT pathway for SAM regeneration. The role of Y97_(Bs)_ in SPL catalysis needs further elucidation and is, thus, not shown here (the figure is adapted with permission from reference [67]. Copyright (2013) American Chemical Society).

The structural assignments above are partially supported by the reaction stoichiometry between the SAM consumed and the SP repaired. As mentioned above, it was suggested by Cheek *et al.* that one molecule of SAM can support >500 turnovers in SPL catalysis using SP containing plasmid DNA as substrate [56]. As shown by Yang *et al*., using dinucleotide SP TpT as substrate, one SAM only supports ~1.5 turnovers (Table 1), suggesting only one third of SAM is regenerated [65]. Such a low SAM regeneration ratio is tentatively ascribed to the weak binding affinity of the dinucleotide SP, which subsequently weakens the SAM/5′-dA binding to the enzyme [65,67,69]. In contrast, the C141A_(Bs)_ and Y99F_(Bs)_ (equivalent of the Y98F_(Gt)_) mutations are expected to disrupt the putative radical transfer chain, resulting in no SAM regeneration. As a consequence, the amount of 5′-dA obtained is equal to the amount of SP repaired (TpT formed); one molecule of SAM is consumed after each catalytic turnover (Table 1). Interestingly, despite its potential role in catalysis, the 5′-dA/TpT ratio was found to be 1.6 ± 0.2 in the Y97F_(Bs)_ reaction, which is identical to that found with the WT SPL. This observation is consistent with the proposed SPL mechanism in Figure 7, which indicates that Y97_(Bs)_ is not a direct component of the radical transfer chain.

Taken together, current experimental findings suggest that the SPL catalyzed SP repair reaction contains a radical transfer pathway, which is composed of four hydrogen atom transfer (HAT) steps (1–4, Figure 7). During the first HAT step, the 5′-dA• generated after the SAM cleavage reaction abstracts the H*_6proR_* atom to yield a SP radical, which fragments to a thymine methyl radical. The radical takes an H atom from C141 in *B. subtilis* SPL in the second HAT process, yielding a thiyl radical. The third HAT step produces the Y99•, which abstracts an H-atom from 5′-dA in the last HAT step to regenerate the 5′-dA•. It then combines with methionine to regenerate SAM after each catalytic cycle.

## 7. Summary and Outlook

The experimental data obtained to date strongly implicate SPL to be the first member of the radical SAM superfamily to bear a radical transfer chain. This transfer chain is essential for a potential SAM regeneration. Currently, only SPL, lysine-2,3-aminomutase and 7-carboxy-7-deazaguanine synthase have been shown to use SAM catalytically [34,39,80], though more are expected, considering the large number of radical SAM enzymes discovered to date (more than 44,000) [38].

Despite the breakthroughs in the past five years, a lot of major issues still remain with this SPL enzyme. For instance, despite that five radical intermediates are indicated in Figure 7, with almost all of them being fairly stable, none has been characterized by EPR spectroscopy. It is likely that the radical transfer steps are tightly coupled, making these radicals transient species. Although a recent report by Kneuttinger *et al.* suggested that the •Y98_(Gt)_ may be observable at the SPL_(Gt)_ “steady state” in a UV-visible difference spectrum [63], that spectrum did not resemble the sharp-peak absorbance exhibited by a typical •Y [81–84]. The cause of the spectral difference may be ascribed to the FeS chromophores, rather than to any tyrosyl radical [67]. Future effort using rapid enzyme kinetic techniques, such as the rapid freeze quench coupled by EPR spectroscopy, are probably needed to trap and characterize these putative radical intermediates.

Furthermore, the substrates used for current enzymology studies are dinucleotide and dinucleoside SPs. Comparing with the SPs in spore genomic DNA, these minimum substrates lack the negatively charged phosphodiester groups upstream and downstream of the SP damage to have a strong binding interaction with the enzyme. The weak binding affinity of these short substrates may drastically reduce the binding affinity of SAM, making it exchangeable with the excess SAM in media. Consequently, SAM regeneration by the end of the catalytic cycle is no longer necessary. It was found that UV irradiation of a 35 bp duplex oligonucleotide in a dry film under 10% relative humidity resulted in a clean formation of SP [27]. Recently, SP incorporation into oligonucleotide via solid state synthesis using a SP phosphoramidite became possible [85]. Future research using SP containing duplex oligonucleotide to re-examine the SP repair by WT and SPL mutants is needed to shed light on the SP repair process in germinating spores.

## Figures and Tables

**Table 1:** Summary of the *Bs* SPL reactions.

SPL enzyme	*v* (min^−1^)	Apparent KIE	Competitive KIE	SP repaired/5′-dA
W.T.	0.41 ± 0.03	2.8 ± 0.3	3.4 ± 0.3	1.5 ± 0.2
C141A_(Bs)_	0.14 ± 0.02	1.7 ± 0.2	3.0 ± 0.3	1.08 ± 0.1
Y97,99A_(Bs)_	N.A.	N.A.	N.A.	N.A.
Y97F_(Bs)_	0.12 ± 0.01	16 ± 1.5	11.5 ± 1.5	1.6 ± 0.2
Y99F_(Bs)_	0.06 ± 0.005	10.5 ± 1	9 ± 1	1.0 ± 0.1
Y97,99F_(Bs)_	<0.004	N.A.	N.A.	0.92 ± 0.1

Note: N.A. = not available.
